# Influence of sports expertise level on attention in multiple object tracking

**DOI:** 10.7717/peerj.5732

**Published:** 2018-09-28

**Authors:** Fanghui Qiu, Yanling Pi, Ke Liu, Xuepei Li, Jian Zhang, Yin Wu

**Affiliations:** 1 School of Kinesiology, Shanghai University of Sport, Shanghai, China; 2 Shanghai Punan Hospital of Pudong New District, Shanghai, China; 3 School of Economics and Management, Shanghai University of Sport, Shanghai, China

**Keywords:** Visual attention, Sports expertise, Multiple object tracking

## Abstract

**Background:**

This study aimed to investigate whether performance in a multiple object tracking (MOT) task could be improved incrementally with sports expertise, and whether differences between experienced and less experienced athletes, or non-athletes, were modulated by load.

**Methods:**

We asked 22 elite and 20 intermediate basketball players, and 23 non-athletes, to perform an MOT task under three attentional load conditions (two, three, and four targets). Accuracies were analyzed to examine whether different levels of sports expertise influence MOT task performance.

**Results:**

The elite athletes displayed better tracking performance compared with the intermediate or non-athletes when tracking three or four targets. However, no significant difference was found between the intermediate athletes and the non-athletes. Further, no differences were observed among the three groups when tracking two targets.

**Discussion:**

The results suggest that the effects of expertise in team ball sports could transfer to a non-sports-specific attention task. These transfer effects to general cognitive functions occur only in elite athletes with extensive training under higher attentional load.

## Introduction

Elite athletes display superior visual attention performance compared to novices/non-athletes (Alves et al., 2013; Heppe et al., 2016; Verburgh et al., 2014; Wang et al., 2015). The ability to track multiple objects plays a critical role in team ball sports situations (Cavanagh & Alvarez, 2005; Cavanagh, Battelli & Holcombe, 2014; Faubert & Sidebottom, 2012; Memmert, 2009; Memmert, Simons & Grimme, 2009). For example, when playing basketball, players are required to track the ball while simultaneously monitoring the movements and positions of their teammates and opponents on the court. To explore this dynamic, spatiotemporal attention in the laboratory, researchers have employed the multiple object tracking (MOT) task (Pylyshyn & Storm, 1988). Previous studies have demonstrated that elite athletes exhibit improved ability relative to novices in tracking multiple objects (Mangine et al., 2014), and training using the MOT task could improve sports performance (Junyent et al., 2015; Romeas, Guldner & Faubert, 2016). Using a 3-D MOT task, Mangine et al. (2014) found that basketball players showed an ability to track multiple objects at higher speeds compared with novices. Romeas, Guldner & Faubert (2016) found that the laboratory training using a 3-D MOT task led to improvements in passing decision-making in a live game situation. However, it remains unclear whether performance in an MOT task could be improved incrementally with sports expertise, and whether differences between experienced athletes, and less experienced athletes or non-athletes were modulated by load.

It has been suggested that an enriched environment leads to plastic changes in the brain (Colcombe et al., 2004; Kempermann, Kuhn & Gage, 1997; May, 2011; Rosenzweig & Bennett, 1996). The sports environment may represent such an enriched setting entailing physical and mental challenges (Alves et al., 2013). One hypothesis is that transfer can occur if the training and transfer tasks engage overlapping cognitive processes and brain regions (Dahlin et al., 2008; Jonides, 2004). The MOT task is a popular paradigm for investigating goal-driven attention in dynamic environments, which is used to assess several aspects of attention, including selective, distributed, and sustained attention skills (Faubert, 2013; Faubert & Sidebottom, 2012; Koldewyn et al., 2013; Memmert, Simons & Grimme, 2009; Scholl, 2009). The MOT paradigm closely replicates the demands of the team ball sports situation (Faubert, 2013; Faubert & Sidebottom, 2012; Mangine et al., 2014; Scholl, 2009). It may be reasonable to suggest that professional team ball players exhibit superior MOT performances owing to extensive sports training (Alves et al., 2013).

Multiple object tracking is accomplished by maintaining a focus of attention over each target as it moves (Cavanagh & Alvarez, 2005). According to the flexible resource theory (Alvarez & Franconeri, 2007; Suchow et al., 2014; Tullo, Faubert & Bertone, 2018), performance in the MOT task is governed by the limitations of a shared resource that can be flexibly allocated to tracked objects, and this is dependent upon task demands, such as the number of target objects. As the number of targets increases, the amount of attentional resources allocated to each target decreases, leading to a decrease in tracking performance (Alvarez & Franconeri, 2007; Culham, Cavanagh & Kanwisher, 2001; Drew, Horowitz & Vogel, 2013; Jovicich et al., 2001; Ma & Flombaum, 2013; Shim et al., 2010; Tomasi et al., 2007). When the number of targets increases to a certain extent, it would be beyond the upper limit of attentional resource to achieve the same level of performance, making the distinction between athletes and non-athletes more measurable. Therefore, the influence of sports expertise on the ability to track multiple objects may be modulated by number of targets.

This study focused on basketball to investigate the relationship between performance in an MOT task and sports expertise. A group of elite basketball players, a group of intermediate basketball players, and a group of non-athletes were recruited to perform an MOT task under three attentional load conditions (two, three, and four targets). Accuracies were analyzed to examine whether different levels of sports expertise influence performance in an MOT task. We hypothesized that elite athletes would exhibit superior MOT task performance owing to extensive sports training (Alves et al., 2013). Moreover, we hypothesized that the sports expertise effects in terms of dynamic attention were modulated by attentional load; that is, differences between groups would be greater in the more difficult condition.

## Materials and Methods

### Participants

The subjects comprised 22 elite basketball players (mean age: 20.68 ± 1.39 years, range: 18–24 years), 20 intermediate basketball players (mean age: 20.20 ± 2.35 years, range: 18–26 years), and 23 age- and education-matched non-athletes (mean age: 20.39 ± 2.29 years, range: 18–27 years). All participants were right-handed men. The elite basketball players were national first- and second-level athletes, recruited from the basketball team of Shanghai University of Sport. They trained an average of 14.50 ± 2.99 h per week (range: 10–20 h) for 6.68 ± 1.47 years (range: 5–10 years). The intermediate basketball players were physical education students majoring in basketball at the Shanghai University of Sport. They trained an average of 6.33 ± 1.60 h per week (range: 4–10 h) for 2.90 ± 0.79 years (range: 2–4 years). The non-athletes were university students without experience in professional basketball training or any other sports. All participants reported normal or corrected-to-normal vision. The participants were asked to refrain from consuming caffeine-containing drinks or alcohol for at least 24 h prior to the experiment. They were also asked not to stay up late to ensure quality of sleep and not to exercise vigorously to ensure a good condition for the experiment. The experimental protocol was approved by the regional ethics committee of the Shanghai University of Sport (No. 2014024SUS). All participants provided written informed consent prior to the start of the experiment.

### Stimuli

**Figure 1:**
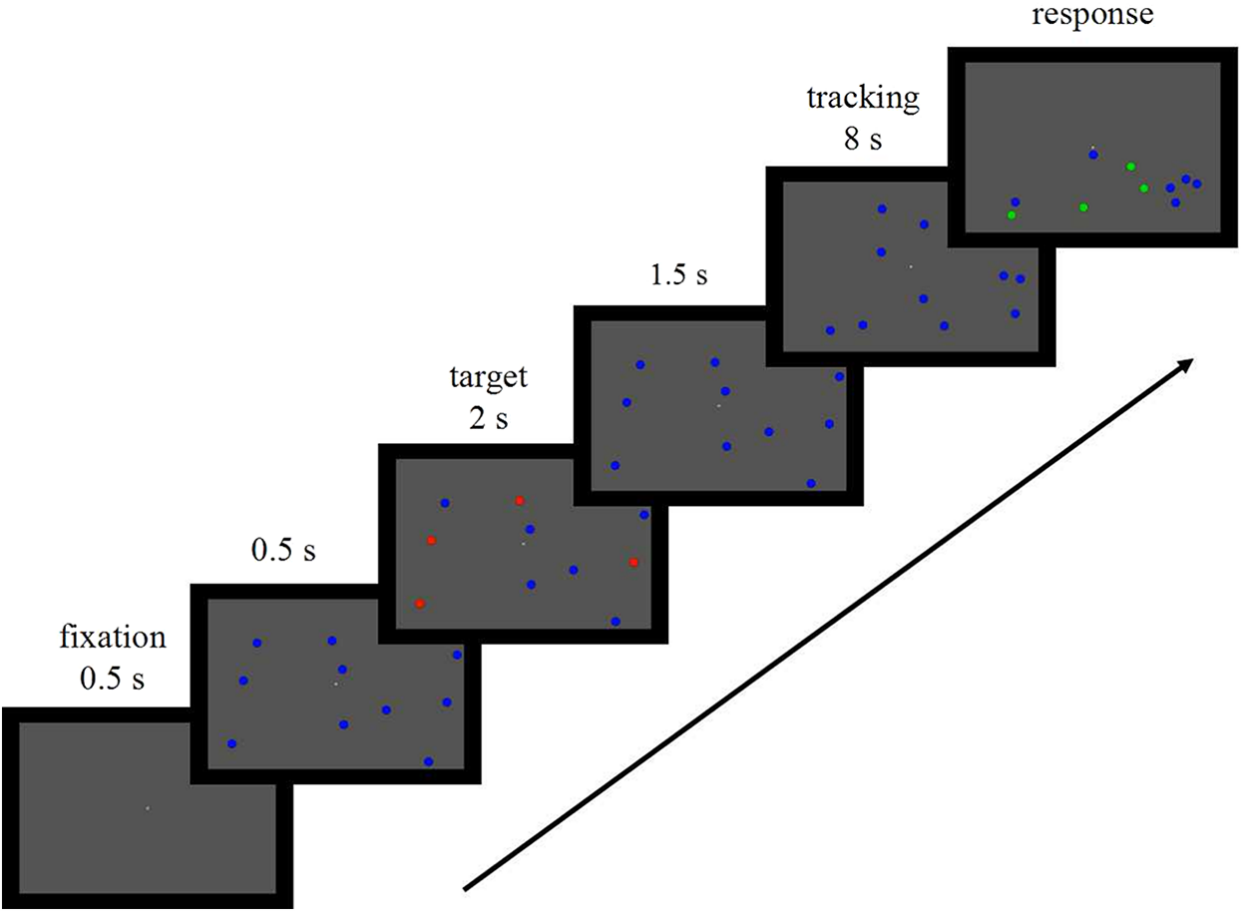
A schematic diagram of the visual stimulus in the multiple object tracking task. Ten blue objects were presented and then a subset of the objects (2, 3, or 4) were highlighted in red for 2 s before turning back to blue. After 1.5 s, all the objects moved at 6.88°/s for 8 s. At the end of the tracking period, the objects stopped moving and the participant were requested to indicate the number of the probe items that matched the tracked items.

The MOT task was first introduced by Pylyshyn & Storm (1988). The stimuli and procedure in the current experiment are based on their previous studies (Pylyshyn, 1989; Pylyshyn, 2006; Pylyshyn & Annan, 2006; Pylyshyn & Storm, 1988). The experiment was run on a 2.5 GHz Lenovo laptop running Windows 7. Visual stimuli were created using MATLAB and were presented on the computer screen. Stimuli were displayed on a 15.1 inch (50.33 cm) LCD monitor. The monitor had a resolution of 1920 × 1080 pixels and a refresh rate of 60 Hz. Participants responded to stimuli using a standard keyboard. The experiment was conducted in a quiet, lit room. Participants were seated approximately 50 cm from the screen. For each trial, a white fixation point was first presented for 0.5 s on a gray background (24.75° × 16.39°), followed by 10 blue objects (diameter 0.92°) for 0.5 s. Then a subset of the objects (two, three, or four) was highlighted in red for 2 s to designate them as targets for the tracking task. Next, the targets were turned back to blue so that no cue remained to distinguish them from the untracked items. After 1.5 s, all 10 objects started to move in random directions at a constant speed (6.88°/s) for 8 s. Finally, at the end of the tracking period, the objects stopped moving before a subset of the objects (the same number of previous targets in the same trial) turned green (probe; Fig. 1). The participants were instructed to press a number key to indicate the number of probe items that matched the targets. Initial object positions were generated randomly from trial to trial. Objects made random changes per second to make the object movements unpredictable. To avoid collision or overlap, the objects were programmed to change direction of movement when approaching each other or touching the screen border.

### Procedure

The experiment consisted of 72 trials divided into 24 cycles. Each cycle contained three trials (two, three, and four targets). Each trial was presented in pseudorandom order across the cycles. Before the formal experiment, six practice trials were conducted. Based on previous studies (Memmert, Simons & Grimme, 2009; Pylyshyn, 2004, 2006; Pylyshyn & Annan, 2006), to avoid an unexpected influence of quick response on tracking accuracy, we did not ask the participants to respond quickly to ensure accuracy.

### Data analysis

Accuracies were analyzed using the two-way analysis of variance (ANOVA) with group (elite players, intermediate players, and non-athletes) as a between-subject factor, and attentional load (two, three, and four targets) as a within-subject factor. We used the Statistical Package for the Social Sciences for Windows version 22.0 for the statistical analyses.

## Results

The participants’ tracking task accuracy averaged 83.97 ± 7.37% for two targets, 59.61 ± 12.80% for three targets, and 45.19 ± 13.12% for four targets. The 3 × 3 two-way ANOVA on accuracy data revealed a significant main effect for group, *F*(2, 62) = 9.57, *p* < 0.001, η^2^ = 0.24, and attentional load, *F*(2, 124) = 329.70, *p* < 0.001, η^2^ = 0.84. More importantly, a significant interaction was observed between group and attentional load, *F*(4, 124) = 3.95, *p* = 0.005, η^2^ = 0.11), suggesting that the latter modulated the group differences.

**Figure 2:**
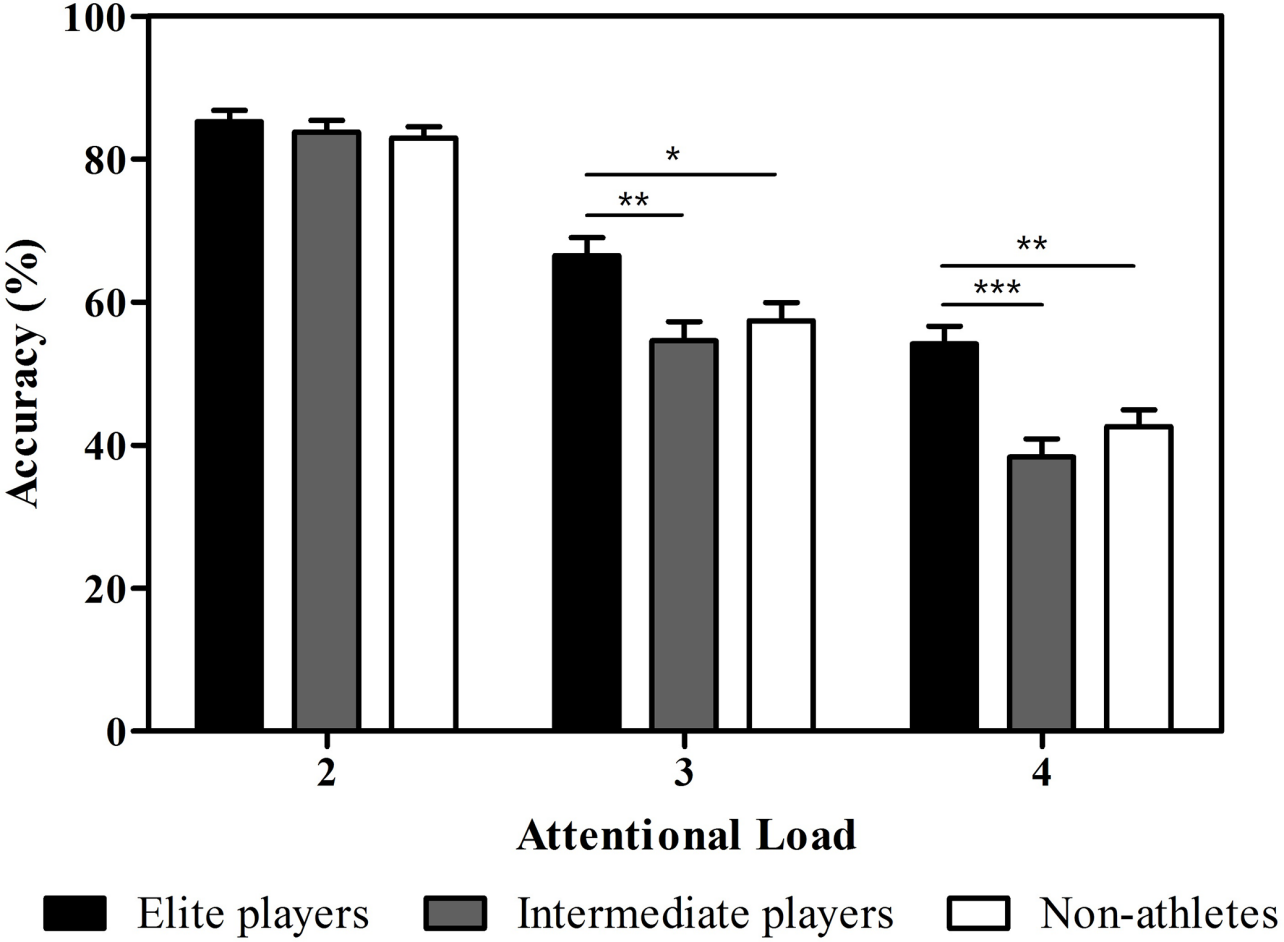
Behavioral performance as a function of the attentional load (number of targets). Error bars represent ±1 standard error of the mean. *p < 0.05, **p < 0.01, ***p < 0.001.

Post hoc analyses indicated that tracking accuracies among the elite players were higher than those of the intermediate players or non-athletes. Moreover, accuracies decreased as tracking load increased for all three groups. More specifically, we found that accuracy scores were higher for the elite players (66.48 ± 14.35%) than either the intermediate players (54.58 ± 12.67%, *p* = 0.006) or the non-athletes (57.43 ± 8.14%, *p* = 0.041) in tracking three targets, *F*(2, 62) = 5.80, *p* = 0.005, η^2^ = 0.16. Similarly, the elite players’ accuracies (54.17 ± 14.09%) were higher than those for either the intermediate players (38.33 ± 10.35%, *p* < 0.001) or the non-athletes (42.57 ± 9.40%, *p* = 0.004) in the tracking of four targets, *F*(2, 62) = 10.93, *p* < 0.001, η^2^ = 0.26. However, intermediate players’ performance did not differ from that of the non-athletes when tracking three or four targets (*p* > 0.5). There were no significant differences among elite players (85.23 ± 8.60%), intermediate players (83.75 ± 7.27%), and non-athletes (82.97 ± 6.27%) in tracking two targets (*F*(2, 62) = 0.53, *p* = 0.59). Figure 2 shows the tracking accuracies of the three groups as a function of attentional load.

## Discussion

This study aimed to investigate the relationship between levels of team ball sports expertise and MOT task performance. Our results showed that elite athletes displayed improved tracking performance compared with intermediate athletes or non-athletes under a high task difficulty condition. Meanwhile, no significant difference was observed between the intermediate athletes and the non-athletes. This finding indicates that team ball sports training is associated with enhanced visual attention ability. This perceptual-cognitive advantage in athletes was modulated by attentional load.

### Expertise-related differences in tracking three or four targets

We found that tracking accuracies of elite athletes were markedly better than the other two groups when the number of targets is three or four, suggesting that the effects of sports expertise transferred from a sport-specific to a general cognitive domain (Alves et al., 2013). This finding is consistent with the evidence in the literature regarding faster visual tracking speed in attentively tracking multiple moving objects in team ball athletes (Faubert, 2013; Mangine et al., 2014) and enhanced visual attention ability in elite athletes (Alves et al., 2013; Hüttermann, Memmert & Simons, 2014; Heppe et al., 2016; Memmert, 2009; Milton et al., 2007; Sanchez-lopez, Silva-pereyra & Fernandez, 2016; Verburgh et al., 2014; Voss et al., 2010). Basketball games involve not only monitoring the speed and direction of the ball but also tracking the positions of teammates and opponents on the court (Faubert, 2013; Faubert & Sidebottom, 2012; Mangine et al., 2014; Scholl, 2009). These tracking processes are very similar to the MOT task. This result supports the hypothesis that a basis for transfer is that training and transfer tasks recruit overlapping cognitive processes (Dahlin et al., 2008; Jonides, 2004).

### Interaction of group with attentional load

An interaction of group with attentional load was observed, suggesting that group differences (i.e., expertise effects) were modulated by attentional load. According to the flexible resource theory (Alvarez & Franconeri, 2007; Suchow et al., 2014; Tullo, Faubert & Bertone, 2018), tracking is mediated by a finite attentional resource that is distributed among the targets. When tracking three or four targets, the limited and flexible resources allocated to each target were fewer among the elite athletes than either the intermediate athletes or the non-athletes, thereby conserving attention for additional objects. By contrast, the three groups did not show any differences in tracking performance when tracking two targets. The lack of difference may be due to the possibility that adequate resources could be devoted to the targets for each group to process the information precisely. Similarly, Memmert, Simons & Grimme (2009) failed to find a significant relationship between team ball sports expertise and tracking performance, which could be attributed to lower difficulty (tracking three targets among seven objects). Future studies should consider task difficulty in comparing experts and non-experts.

### Non-significant difference between intermediate athletes and non-athletes

We found that the intermediate athletes and non-athletes did not differ from each other in terms of tracking accuracies. This finding is consistent with the existing evidence that intermediate players are no better at visual tracking speed than non-athletes before a training session (Faubert, 2013). Similar results were also observed in perceptual and anticipation tasks (Abernethy, Neal & Koning, 1994; Müller et al., 2010). In the present study, the intermediate athletes were trained less frequently for fewer years than the elite athletes. It may be the case that performance on the MOT task may not be improved incrementally with sports expertise and that the transfer from cognitive skills developed during sports training to the general cognitive process occurs only among elite athletes with extensive training. The present study suggested that visual attention ability could not be improved through training less frequently for only two or three years. A longitudinal design is necessary to determine the needed length of sports training to achieve higher levels of visual attention performance.

### Limitations

A few limitations must be taken into consideration. The temporal resolution of dynamic attention is known to limit the attentional tracking of multiple objects (Holcombe & Chen, 2013; Roudaia & Faubert, 2017). Dynamic temporal resolution decreases as the number of targets increases. In the current study, we used a fixed speed for the MOT task. It is possible that the lack of difference between the groups observed for the two target condition may be reflective of the fact that the speed chosen here may be well within the temporal frequency limit. Therefore, it is theoretically possible that differences between groups may be observed if speed thresholds were used in the two target condition. We could not make this distinction using the present design. Future studies with a full design are needed to address this unknown. Another limitation is that we did not ask the participants to respond quickly. The use of the time-pressured response, which is similar to real-life situations, may be more likely to yield differences between the groups. The untimed response, which was used to ensure accuracy, might instead lead to an extensive decision process that obscured possible differences in tracking two targets. In addition, although these differences were insignificant, the intermediate athletes’ accuracies were slightly lower than those of the non-athletes in the tracking of three and four targets. It is possible that individual differences may lead to the intermediate athletes’ reduced performance relative to that of the non-athletes.

## Conclusion

This study suggested that the effects of expertise on team ball sports could transfer to non-sports-specific domains; moreover, the transfer effects occur only among elite athletes with extensive training under a higher attentional load. These findings provide valuable information that will help fill the gap in understanding the transfer effects of sports training on non-sports-specific visual attention ability improvement.

## Supplemental Information

10.7717/peerj.5732/supp-1 Supplemental Information 1 Raw data.

## Additional Information and Declarations

### Data Availability

The following information was supplied regarding data availability: The raw data are provided in Data S1.
